# The bHLH transcription factor GmPIB1 facilitates resistance to *Phytophthora sojae* in *Glycine max*

**DOI:** 10.1093/jxb/ery103

**Published:** 2018-03-22

**Authors:** Qun Cheng, Lidong Dong, Tianjiao Gao, Tengfei Liu, Ninghui Li, Le Wang, Xin Chang, Junjiang Wu, Pengfei Xu, Shuzhen Zhang

**Affiliations:** 1 Soybean Research Institute/Key Laboratory of Soybean Biology of Chinese Education Ministry, Northeast Agricultural University, Harbin, China; 2 School of Life Sciences, Guangzhou University, Guangzhou, China; 3 Jiamusi Branch Academy of Heilongjiang Academy of Agricultural Sciences, Jiamusi, China; 4 Soybean Research Institute of Heilongjiang Academy of Agricultural Sciences, Key Laboratory of Soybean Cultivation of Ministry of Agriculture P. R. China, Harbin, China

**Keywords:** bHLH transcription factor, *Glycine max*, *Phytophthora sojae*, root, ROS

## Abstract

*Phytophthora sojae* Kaufmann and Gerdemann causes Phytophthora root rot, a destructive soybean disease worldwide. A basic helix–loop–helix (bHLH) transcription factor is thought to be involved in the response to *P. sojae* infection in soybean, as revealed by RNA sequencing (RNA-seq). However, the molecular mechanism underlying this response is currently unclear. Here, we explored the function and underlying mechanisms of a bHLH transcription factor in soybean, designated GmPIB1 (*P. sojae*-inducible bHLH transcription factor), during host responses to *P. sojae*. *GmPIB1* was significantly induced by *P. sojae* in the resistant soybean cultivar ‘L77-1863’. Analysis of transgenic soybean hairy roots with elevated or reduced expression of *GmPIB1* demonstrated that GmPIB1 enhances resistance to *P. sojae* and reduces reactive oxygen species (ROS) accumulation. Quantitative reverse transcription PCR and chromatin immunoprecipitation–quantitative PCR assays revealed that GmPIB1 binds directly to the promoter of *GmSPOD1* and represses its expression; this gene encodes a key enzyme in ROS production. Moreover, transgenic soybean hairy roots with *GmSPOD1* silencing through RNA interference exhibited improved resistance to *P. sojae* and reduced ROS generation. These findings suggest that GmPIB1 enhances resistance to *P. sojae* by repressing the expression of *GmSPOD1*.

## Teaser

GmPIB1, previously reported to be up-regulated in *Rps* near-isogenic lines, enhances resistance to *Phytophthora sojae* in soybean by repressing expression of *GmSPOD1*, a key enzyme for producing reactive oxygen species.

## Introduction

Phytophthora root and stem rot caused by *Phytophthora sojae* is one of the most destructive soybean diseases worldwide, resulting in annual losses of $1–2 billion globally (Tyler, 2007). The most economical and effective way to protect soybeans against *P. sojae* infection is by breeding for dominant resistance to *P. sojae* (*Rps*) genes (Sugimoto *et al.*, 2012). However, the continuous utilization of a single *Rps* gene can result in selective pressure that promotes the evolution of more pathogenic races of *P. sojae*. Thus, a particular *Rps* gene is effective for only 8–15 years (Walker and Schmitthenner, 1984; Tooley and Grau, 1984; Sugimoto *et al.*, 2012). Moreover, some genes encode proteins that most likely function in direct protection, such as key enzymes for osmolyte biosynthesis, antioxidant and reactive oxygen species (ROS) scavengers, and enzymes involved in many metabolic processes (Yan *et al.*, 2014; Cheng *et al.*, 2015; Wang *et al.*, 2015*a*; Yan *et al.*, 2016). The products of regulatory genes, including membrane-localized receptors, calcium sensors, kinases, and transcription factors (TFs), participate in further signal transduction and the regulation of gene expression (Wang *et al.*, 2015*a*). Several TF families play important roles in plant stress tolerance, such as basic helix–loop–helix (bHLH), DREB, ERF, WRKY, MYB, bZIP, and NAC TFs (Tran *et al.*, 2004; Hu *et al.*, 2006; Kim and Kim, 2006; Liao *et al.*, 2008*a*,b; Zhou *et al.*, 2008; Seo *et al.*, 2010; Hao *et al.*, 2011; Niu *et al.*, 2012; Liu *et al.*, 2014; Dong *et al.*, 2015). These TFs separately or cooperatively affect the expression of various downstream genes and constitute gene networks for stress adaptation (Wang *et al.*, 2015*a*).

Members of the bHLH family, which are distinguished by the bHLH domain, are universally found in eukaryotes (Duek and Fankhauser, 2005; Liu *et al.*, 2014). The bHLH domain consists of 50–60 amino acids with two functionally distinct regions: the basic region (containing 13–17 primarily basic amino acids for DNA binding) and the HLH region (which enables the formation of homodimers or heterodimers with one or several different partners) (Toledo-Ortiz *et al.*, 2003; Feller *et al.*, 2011). The bHLH TFs are involved in essential plant physiological and developmental processes by binding to E-box (CANNTG)/G-box (CACGTG) sequences in the promoters of stress-response genes (Kim and Kim, 2006; Liu *et al.*, 2013; Liu *et al.*, 2014). For instance, CIB1 is a bHLH TF that binds to the G-box DNA motif *in vitro* but heterodimerizes with other CIB1-related proteins that in turn bind to E-box sequences to regulate transcription *in vivo* (Liu *et al.*, 2013). bHLH122 binds directly to the G-box/E-box *cis*-elements in the *CYP707A3* promoter and represses its expression, and *bHLH122* is strongly induced by drought, NaCl, and osmotic stress in Arabidopsis (Liu *et al.*, 2014). Increasing evidence indicates that bHLHs regulate plant responses to biotic and abiotic stresses (Zhang *et al.*, 2011; Liu *et al.*, 2014; Wang *et al.*, 2015*b*; Turnbull *et al.*, 2017). For example, phytochrome-interacting factor 4 (PIF4), a nucleus-localized bHLH protein, interacts directly with brassinazole-resistant 1 (BZR1) and forms a module that integrates steroid and environmental signaling (Oh *et al.*, 2012). Abscisic acid (ABA)-inducible bHLH TF/jasmonic acid (JA)-associated MYC2-like 1 (JAM1), a repressor of JA signaling, plays a pivotal role in the ﬁne-tuning of JA-mediated stress responses and plant growth (Nakata *et al.*, 2013). ABA-inducible gene (*AtAIG1*), encoding a bHLH-type TF in Arabidopsis, is up-regulated after exposure to ABA but not to cold or NaCl, suggesting that AtAIG1 might be involved in ABA-mediated responses (Kim and Kim, 2006). *ICE1*, which is constitutively expressed in Arabidopsis, encodes a bHLH TF that regulates the expression of CBF genes in response to cold stress (Chinnusamy *et al.*, 2003; Lee *et al.*, 2005). Overexpressing *OrbHLH001* improves freezing and salt tolerance in Arabidopsis. Moreover, the Arabidopsis bHLH TF HBI1 is a negative regulator of the basal defense response. Loss-of-function of *HBI1* increases resistance to bacterial infection, and constitutive overexpression of *HBI1* reduces pathogen-associated molecular pattern (PAMP)-induced immune responses (Fan *et al.*, 2014). The transient overexpression of *StCHL1* significantly increases leaf colonization of *Nicotiana benthamiana* by *P. infestans*, which is consistent with the finding that its homologs, HBI1 and CIB1, are negative regulators of immunity responses (Turnbull *et al.*, 2017). However, the potential functions of most bHLH family members in soybean are still unclear.

A bHLH TF gene was shown to be up-regulated in all 10 near-isogenic lines (NILs) examined, each with a unique *Rps* gene/allele, based on sequencing and comparative transcriptome analysis of the NILs and the susceptible parent ‘Williams’ pre- and post-inoculation with *P. sojae* (Lin *et al.*, 2014). Therefore, in the current study, we isolated this bHLH TF gene from *P. sojae*-resistant soybean cultivar ‘L77-1863’, which we designated *GmPIB1* (*P. sojae-inducible bHLH transcription factor*; Glyma.01g129700). Overexpressing *GmPIB1* in transgenic soybean hairy roots increased resistance to *P. sojae*, whereas RNA interference (RNAi) of this gene in transgenic soybean hairy roots increased susceptibility to this pathogen. GmPIB1 bound directly to the promoter of *GmSPOD1* and inhibited its expression, leading to improve resistance to *P. sojae*. Taken together, these results indicate that GmPIB1 facilitates the resistance response of soybean to *P. sojae* infection by repressing the expression of *GmSPOD1*.

## Materials and methods

### Plant material, treatments, and primers

The *P. sojae*-susceptible soybean cultivar ‘Williams’ (*rps*1b) and the resistant cultivar ‘L77-1863’ (*Rps*1b) (Shan *et al.*, 2004) were used in this study. The seeds were sown in pots in a growth chamber maintained at 25 °C and 70% relative humidity with a 16 h light/8 h dark cycle. Fourteen days after planting, seedlings at the first-node stage (V1; Fehr *et al.*, 1971) were subjected to various treatments.

For abiotic treatments, ‘L77-1863’ plants were exposed to one of three diﬀerent hormones, namely, methyl jasmonate (MeJA), ethylene (ET), or salicylic acid (SA). SA (2 mM) and MeJA (100 µM) were dissolved in 0.01% Tween 20 and sprayed onto young leaves for 0, 1, 3, 6, 9, 12, or 24 h. Ethylene treatment was performed by injecting gaseous ethylene at a concentration of 200 µl l^−1^ into a sealed Plexiglas chamber for 0, 1, 3, 6, 9, 12, or 24 h. The control leaves were sprayed with an equal volume of 0.01% (v/v) Tween 20.

For *P. sojae* treatment, plants of the susceptible cultivar ‘Williams’ and the resistant cultivar ‘L77-1863’ were inoculated with *P. sojae* race 1 (Zhang *et al.*, 2010) zoospores as described by Ward *et al.* (1979). Unifoliate leaves were treated for 0, 6, 9, 12, 24, 36, 48, or 72 h. The susceptible soybean cultivar ‘Williams’ and resistant cultivar ‘L77-1863’ were obtained from the Key Laboratory of Soybean Biology at the Chinese Ministry of Education, Harbin, and used for the gene transformation experiments. All primers used for vector construction, PCR, and quantitative reverse transcription (qRT)-PCR assays for all target genes are listed in Supplementary Table S1 at *JXB* online.

### RT-PCR and qRT-PCR analysis

Total RNA was isolated from ‘Williams’ and ‘L77-1863’ soybean leaves using Trizol reagent (Invitrogen, Shanghai, China). cDNA synthesis was conducted using an M-MLV reverse transcriptase kit (Takara, Dalian, China) according to the manufacturer’s instructions. RT-PCR was performed to analyse *GmPIB1* transcript levels in ‘Williams’ and ‘L77-1863’ plants according to Zhang *et al.* (2012). The soybean housekeeping gene *GmEF1β* (GenBank accession no. NM_001248778) was used as the internal control. qRT-PCR analysis was performed to measure *GmPIB1* transcript levels on a CFX96 Touch™ Real-Time PCR machine (Bio-Rad, USA) using a real-time PCR kit (Toyobo, Japan). The soybean housekeeping gene *GmEF1β* was used as an internal reference to normalize all data. The relative transcript level of the target gene was calculated using the 2^−ΔΔ*C*T^ method. Three biological replications per line were performed in each test.

### Subcellular localization of GmPIB1 fusion protein

The coding sequence of *GmPIB1* was amplified by RT-PCR using primers *GmPIB1GF* and *GmPIB1GR*. The coding sequence was fused to the N-terminus of green fluorescent protein (GFP) under the control of the constitutive CaMV35S promoter. The resulting expression vector, *p35S:GmPIB1-GFP*, was transformed into Arabidopsis protoplasts via polyethylene glycol (PEG)-mediated transfection as described by Yoo *et al.* (2007). Fluorescence signals were imaged using a TCS SP2 spectral confocal microscope imaging system (Leica, Germany). The *p35S:GFP* vector was used as a control.

To analyse the expression of GmPIB1 fusion protein in plants, membrane, nuclear, and cytoplasmic proteins were extracted using a Cytoplasmic, Nuclear, and Membrane Protein Extraction Kit (Sangon Biotech, Shanghai, China, C510002). The supernatants of extracts were separated by SDS-PAGE. After electrophoresis, the proteins were transferred to polyvinylidene difluoride membranes (Millipore) and probed using anti-GFP antibodies (Abmart, M2004).

### Expression and puriﬁcation of fusion protein

The open reading frame of *GmPIB1* was fused to the N-terminus of the 6×His-tag at the *Eco*RI and *Xho*I restriction sites of the vector pET29b(+) (Novagen, Germany). The recombinant fusion plasmid was expressed in *Trans*etta (DE3) *E. coli* cells (TransGen Biotech, China). His-tagged protein production was induced with 0.5 mM isopropyl-β-D-thiogalactoside (IPTG) at 37 °C for 4 h. The fusion protein was puriﬁed at 4 °C according to the pET System Manual (Novagen). The GmPIB1–His fusion protein was subsequently analysed by SDS-PAGE and immunoblotting using an anti-His antibody.

### Electrophoretic mobility shift assay

The DNA-binding activity of GmPIB1 was examined using a digoxigenin-ddUTP-labeled double-stranded oligonucleotide E-box probe as described previously (Meng *et al.*, 2013). The sequence of the probe for the E-box was 5′-AGGAGAGTGGGC*CANNT G*CGCTCTTTTGCATTC-3′ and that of the mutant E-box (mE-box) was 5′-AGGAGAGTGGGC*CCNN CG*CGCTCTTTTGCATTC-3′. The electrophoretic mobility shift assay (EMSA) was performed as described by Kass *et al.* (2000).

### Transactivation assay

For the transactivation assay, the β-glucuronidase (*GUS*) gene in pCAMBIA3301 was replaced by *GmPIB1* as the effector plasmid. The E-box was multimerized four times and placed upstream of the cauliflower mosaic virus (CaMV) 35S promoter (–42 to +8) containing a TATA box. This construct was inserted into pXGUS-P (Chen *et al.*, 2009) and fused to the *GUS* gene as the reporter plasmid. The transactivation assay was performed by PEG transfection of Arabidopsis protoplasts as described by Yoo *et al.* (2007). Twenty micrograms of reporter plasmid and 20 µg of effector plasmid or control plasmid (pXGUS-P-35Smini) were co-transfected into 4 × 10^4^ protoplasts. The transfected cells were incubated at 22 °C in the light for 18–20 h. GUS activity was determined as described (Lu *et al.*, 1998).

### *Agrobacterium rhizogenes*-mediated transformation of soybean hairy roots

To construct the *p35S:GmPIB1-Myc* overexpression vector, the coding sequence of *GmPIB1* with a C-terminal 4×Myc fusion sequence was cloned into plant expression vector pCAMBIA3301 with gene-specific primers. To construct the *GmPIB1* RNAi vector, the cDNA fragment of *GmPIB1* was amplified using the primer set *PIB1RNAi-F/R* and inserted into vector pFGC5941 (Kerschen *et al.*, 2004). Transgenic soybean hairy roots were generated by *A. rhizogenes*-mediated transformation as described by Graham *et al.* (2007) and Kereszt *et al.* (2007) with some modifications. The cotyledons were cut into rough triangles and immediately placed in Petri dishes containing 0.6% agar medium to keep them moist. The cut surface was treated with 20 µl *A. rhizogenes* suspension. The dishes were sealed with Parafilm and placed in an incubator at 25 °C. Transformed hairy roots were abundant along a callus ridge on the inoculated cotyledons after approximately 3 weeks. Overexpression of the target gene in transgenic hairy roots was tested via quantitative PCR (qPCR) and immunoblotting, and RNAi transgenic hairy roots were verified by qPCR and Southern blot analysis.

### Promoter–GUS analysis

The 1494 bp promoter sequence of *GmPIB1* was ampliﬁed using gene-speciﬁc primers *GmPIB1PF* and *GmPIB1PR* and cloned into the pBI121 expression vector. The *GmPIB1* promoter–GUS construct was transformed into the hairy roots of ‘L77-1863’ soybean plants by *A. rhizogenes*-mediated transformation. When the hairy roots generated at the infection site were approximately 8 cm long, the original main roots were treated with *P. sojae* zoospores for 48 h, or MeJA, ET, or SA for 6 h. Soybean hairy roots transformed with empty vector (EV) were used as controls. Histochemical GUS staining was performed 3 h after treatment using GUS staining buffer (1 mM 5-bromo-4-chloro-3-indolyl-b-D-GlcA solution in 100 mm sodium phosphate pH 7.0, 0.1 mM EDTA, 0.5 mM ferrocyanide, 0.5 mm ferricyanide, and 0.1% Triton X-100) at 37 °C overnight. GUS activity was measured as described by Jefferson *et al.* (1987).

### Pathogen response assays of transgenic soybean hairy roots

To investigate whether *GmPIB1*-transformed hairy roots were resistant to pathogen infection, artificial inoculation procedures were performed as described by Ward *et al.* (1979). When the hairy roots generated at the infection site were approximately 8 cm long, the original main roots were incubated with *P. sojae* zoospores in a mist chamber at 25 °C with 100% relative humidity for 2 d. EV soybean hairy roots were used as controls. Disease symptoms on each root were observed after inoculation and photographed with a Nikon B7000 camera.

### *In situ* ROS detection

To investigate whether the *GmPIB1*-transformed soybean hairy roots would respond to oxidative stress, *GmPIB1* transgenic and EV (control) hairy roots were treated with *P. sojae* zoospores for 48 h as described by Ward *et al.* (1979). *In situ* H_2_O_2_ and O_2_^−^ detection were performed using diaminobenzidine (DAB) or Nitro blue tetrazolium (NBT) as described by Lu *et al.* (2011). Total ROS levels were measured according to the instructions supplied with the Reactive Oxygen Species Assay Kit (Beyotime Institute of Biotechnology, Haimen, China). Fluorescence was detected at 485 nm for excitation and 530 nm for emission with a ﬂuorescence microplate reader (Bio-TEK, USA; Qian *et al.*, 2009). Relative ROS levels, i.e. the ratio of total ROS levels in hairy roots under *P. sojae* zoospore versus water treatment (mock) at the same time point were measured.

### Yeast two-hybrid assays

For interaction studies, full-length *GmPIB1* was ampliﬁed using gene-speciﬁc primers *GmPIB1*YF and *GmPIB1*YR and cloned in the pGBKT7 vector and pGADT7 vector. Fusion plasmids pGADT7-GmPIB1 and pGBKT7-GmPIB1 were transformed into yeast strain Y2HGold (Clontech). After selection on SD (−Trp, −Leu) medium, the transformants were transferred to SD (−Trp, −His, −Trp, −Ade) medium to identify protein–protein interactions.

### Bimolecular fluorescence complementation assays

The coding sequence of *GmPIB1* was cloned into serial pSAT6 vectors encoding either N- and C-terminal-enhanced yellow fluorescent protein fragments. The resulting constructs were used for transient assays via PEG transfection of Arabidopsis protoplasts as described by Yoo *et al.* (2007). Transfected cells were imaged using a TCS SP2 confocal spectral microscope imaging system (Leica).

### Chromatin immunoprecipitation–qPCR assays

For chromatin immunoprecipitation (ChIP)–qPCR assays, EV and *p35S:PIB1-Myc* transgenic lines were subjected to chromatin extraction and immunoprecipitation as described by Saleh *et al.* (2008). Briefly, soybean hairy roots were harvested for fixation. Chromatin was isolated and sonicated to generate DNA fragments with an average size of 500 bp. The soluble chromatin fragments were isolated and pre-absorbed with 30 µl Protein G Plus/Protein A Agarose Suspension (Merck Millipore Biotechnology) to eliminate non-specific binding and immunoprecipitated by 30 µl Protein G Plus/Protein A Agarose Suspension with anti-Myc (Santa Cruz Biotechnology). The precipitated DNA was recovered and analysed by qRT-PCR with SYBR Premix ExTaq Mix (Takara Bio). The precipitated and input DNA samples were analysed by qPCR with the gene-specific primers. The data were normalized to input transcript levels and represent the means from three biological replicates.

### Transient expression assay

A transient dual-luciferase assay was performed as previously described (Shang *et al.*, 2010; Song *et al.*, 2013). Briefly, the 1.761 kb promoter sequence of *pGmSPOD1* was cloned using gene-specific primers Gm*SPOD1*P-F/R and inserted into the *Sca*I and *Xba*I sites of the pBI121 vector (Clontech, CA, USA) after its *GUS* gene had been replaced with the firefly luciferase gene. The reporter construct *pGmSPOD1:GUS* and the effector construct *p35S:GmPIB1-Myc* were transformed into *A. rhizogenes* strain K599 and transfected into soybean hairy roots by *A. rhizogenes*-mediated transformation. When the hairy roots generated at the infection site were approximately 8 cm long, the original main roots were stained for GUS. The reporter construct *pGmSPOD1:LUC* and the effector construct *p35S:GmPIB1-Myc* were transformed into *Agrobacterium tumefaciens* strain GV3101 and transfected into healthy 21-day-old *N. benthamiana* tobacco leaves by agroinfiltration as described previously (Liu *et al.*, 2012, Meng *et al.*, 2013). The plants were incubated 3 d after infiltration, sprayed with luciferin (1 mM), and photographed with a CCD camera (Berthold Technologies) at 72 h after infiltration.

### Protein extraction, immunoblotting, and Southern blotting

To analyse protein expression in transgenic plants, total proteins were extracted with protein extraction buffer (50 mM Tris–HCl at pH 7.5, 150 mM NaCl, 5 mM EDTA, 0.1% Triton X-100, and protease inhibitor cocktail (Roche)). Total proteins (200 mg) were separated by SDS-PAGE. After electrophoresis, the proteins were transferred to polyvinylidene difluoride membranes (Millipore) and probed using anti-Myc antibodies (Santa Cruz Biotechnology).

Southern blotting was conducted according to the modified protocol of Zhang *et al.* (2012), in which 20 μg of genomic DNA digested with the restriction enzyme *Hin*dIII was hybridized to a probe derived from the *bar*-specific fragment (354 bp).

## Results

### *GmPIB1* expression is induced upon *P. sojae* infection

**Fig. 1.**
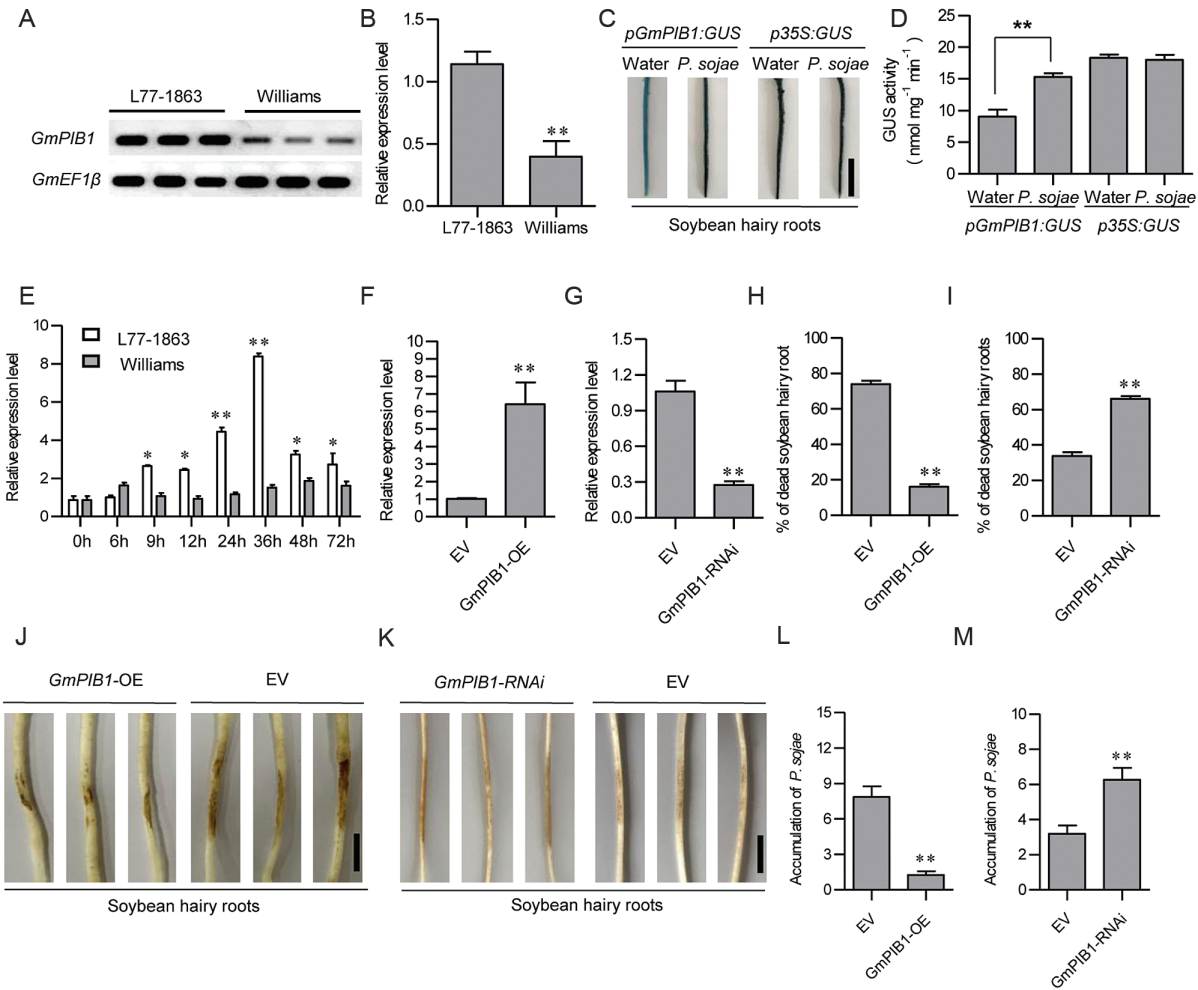
Transcriptional analysis of *GmPIB1*. (A) Expression patterns of *GmPIB1* in susceptible soybean cultivar ‘Williams’ and resistant cultivar ‘L77-1863’, as assessed by RT-PCR. (B) Expression patterns of *GmPIB1* in susceptible cultivar ‘Williams’ and resistant cultivar ‘L77-1863’, as assessed by qPCR. (C) *GmPIB1* promoter-driven *GUS* expression in transgenic soybean hairy roots treated with *P. sojae* or water for 48 h. Bars, 1 cm. (D) GUS activity analysis of *GmPIB1* promoter expression. GUS activity was measured using a 4-methylumbelliferyl-D-glucuronide assay. The data represent the means ±SD of three independent experiments. (E) Relative expression of *GmPIB1* in soybean cultivars ‘Williams’ and ‘L77-1863’ upon *P. sojae* infection. The infected samples were collected at 0, 6, 9, 12, 24, 36, 48, and 72 h after inoculation with *P. sojae* (race 1). Relative *GmPIB1* transcript levels were compared with mock-treated plants at the same time point. (F, G) qRT-PCR analysis of relative *GmPIB1* expression in transgenic soybean hairy roots. Empty vector (EV) transgenic soybean hairy roots were used as controls. (H, I) Percentages of dead EV, *GmPIB1*-OE, and *GmPIB1*-RNAi roots after 5 d of *P. sojae* infection. Each experiment contained at least 50 roots per line, and roots were scored as dead when they were completely rotten. (J, K) Typical infection phenotypes of *GmPIB1*-OE, *GmPIB1*-RNAi, and EV soybean hairy roots after 2 d of *P. sojae* inoculation. Bars, 1 cm. (L, M) Accumulation of *P. sojae* biomass in transgenic soybean hairy roots and EV. *Phytophthora sojae TEF1* (EU079791) transcript levels in infected soybean hairy roots (2 d) were plotted relative to soybean *GmEF1β* (NM_001248778) expression levels, as determined by qRT-PCR. The ampliﬁcation of soybean *GmEF1β* was used as an internal control to normalize all data. The experiment was performed using three biological replicates, each with three technical replicates, and differences were statistically analysed using Student’s *t*-test (*P<0.05, **P<0.01). Bars indicate standard error of the mean. (This figure is available in color at *JXB* online.)

To evaluate whether *GmPIB1* is involved in the response of soybean to *P. sojae* infection, we performed RT-PCR and qRT-PCR to examine the transcript levels of this gene in the susceptible soybean cultivar ‘Williams’ and the resistant cultivar ‘L77-1863’. As shown in Fig. 1A, B, the expression level of *GmPIB1* was much higher in the resistant cultivar ‘L77-1863’ than in the susceptible cultivar ‘Williams’. qRT-PCR assays showed that *GmPIB1* transcript levels were signiﬁcantly elevated and reached a maximum level at 36 h after *P. sojae* treatment in ‘L77-1863’ (Fig. 1D). However, in ‘Williams’, *GmPIB1* transcript levels did not increase under *P. sojae* treatment (Fig. 1E).

 We used the 1494 bp promoter region of *GmPIB1* to drive the expression of the *GUS* reporter gene in the pBI121 expression vector, which we transformed into ‘L77-1863’ soybean hairy roots via high-efﬁciency *A. rhizogenes*-mediated transformation as described by Graham *et al.* (2007) and Kereszt *et al.* (2007). When the hairy roots generated at the infection site were approximately 8 cm long, we subjected the original main roots to gene expression analysis and *P. sojae* treatment. Compared with control roots (treated with water), *GmPIB1* promoter activity was highly induced in roots subjected to *P. sojae* treatment (Fig. 1C, D). Together, these results suggest that GmPIB1 is involved in the defense response of soybean to *P. sojae*.

### Cloning full-length *GmPIB1* cDNA

We then examined whether the *GmPIB1* gene and promoter sequences differ between ‘Williams’ and ‘L77-1863’. We cloned and sequenced the cDNA and promoter of *GmPIB1* in ‘Williams’ and ‘L77-1863’ and found no difference in sequence between the two cultivars (data not shown). *GmPIB1* encodes a deduced 151 amino acid polypeptide with a bHLH domain at amino acid positions 9–63 (see Supplementary Fig. S1A). The predicted three-dimensional model of GmPIB1 consists of two α-helices (Supplementary Fig. S1C). To further explore the evolutionary relationship among plant bHLH proteins, we constructed a phylogenetic tree using MEGA4.0 (Tamura *et al.*, 2007) based on amino acid sequences. Sequence alignment and phylogenetic tree analysis revealed that GmPIB1 shares 65.5–95.2% identity in overall amino acid sequence with bHLH TFs from *Glycine max* (XP_003551597), *Arachis ipaensis* (XP_016186634), *Theobroma cacao* (XP_017974773), *Vigna radiata* var. radiata (XP_014491943), *Vitis vinifera* (XP_002268100), *Gossypium arboretum* (XP_017609785), and *Cicer arietinum* bHLH (XP_004492536) (Supplementary Fig. S1B, D).

### GmPIB1 enhances resistance to *P. sojae* in transgenic soybean hairy roots

To examine the effect of the loss and overexpression of GmPIB1 on resistance to *P. sojae* in soybean, we generated G*mPIB1*-overexpressing (G*mPIB1*-OE) and G*mPIB1*-RNA interference (*GmPIB1*-RNAi) transgenic soybean hairy roots by high-efficiency *A. rhizogenes*-mediated transformation (Graham *et al.*, 2007; Kereszt *et al.*, 2007) in susceptible cultivar ‘Williams’ and resistant cultivar ‘L77-1863’. We examined the *GmPIB1*-OE transgenic hairy roots by immunoblotting (see Supplementary Fig. S2A) and qRT-PCR (Fig. 1F) and the *GmPIB1*-RNAi transgenic hairy roots by Southern blot analysis (Supplementary Fig. S2B) and qRT-PCR (Fig. 1G). As shown in Fig. 1H, ~75% of EV (vector control) transgenic hairy roots of the susceptible cultivar ‘Williams’ inoculated with *P. sojae* were completely dead at 5 d of treatment, whereas only ~18% of inoculated *GmPIB1*-OE transgenic hairy roots were completely dead. However, ~35% of inoculated EV transgenic hairy roots of resistant cultivar ‘L77-1863’ and ~95% of inoculated *GmPIB1*-RNAi transgenic hairy roots were completely dead at 5 d of inoculation with *P. sojae* (Fig. 1I). After 2 d of incubation with *P. sojae* zoospores, the three *GmPIB1*-OE lines displayed almost no visible lesions compared with EV control roots in susceptible cultivar ‘Williams’ (Fig. 1J). By contrast, the three *GmPIB1*-RNAi transgenic hairy root lines exhibits enhanced wilting symptoms and chlorosis compared with EV hairy roots in resistant cultivar ‘L77-1863’ (Fig. 1K).

We also analysed the relative biomass of *P. sojae* in infected soybean hairy roots after 2 d of incubation with *P. sojae* zoospores. The biomass of *P. sojae* (based on the transcript level of *P. sojae TEF1*; GenBank accession no. EU079791) (Blair *et al.*, 2008) was significantly (*P*<0.01) lower in the *GmPIB1*-OE lines than in EV hairy roots (Fig. 1L). However, the biomass of *P. sojae* was significantly (*P*<0.01) higher in the *GmPIB1*-RNAi lines than in EV hairy roots (Fig. 1M). These results indicate that overexpressing *GmPIB1* in soybean hairy roots improves resistance to *P. sojae* and that silencing this gene increases susceptibility to *P. sojae*.

### *GmPIB1* transcript levels under different hormone treatments

**Fig. 2.**
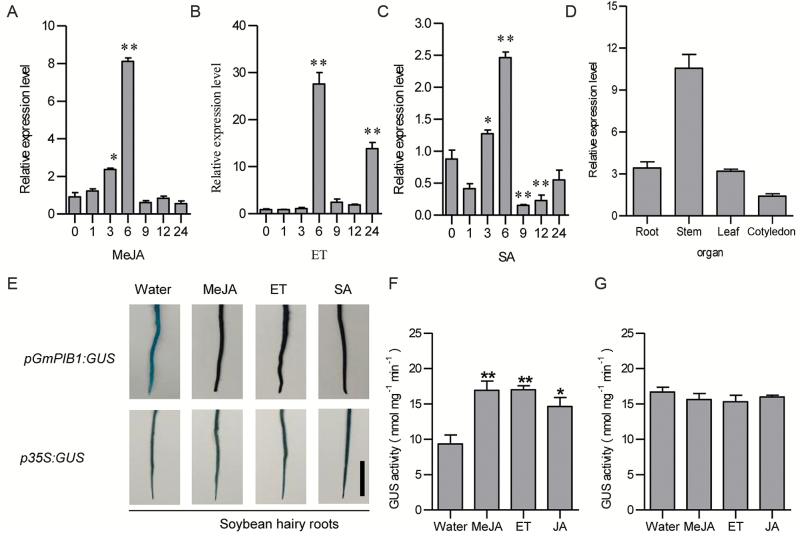
Expression patterns of *GmPIB1* in soybean. (A–C) *GmPIB1* expression in soybean leaves in response to exogenous hormones: 100 μM MeJA, 2 mM SA, and ET treatment for 0, 1, 3, 6, 9, 12, and 24 h. Fourteen-day-old plants were used for treatments and analyses. Relative *GmPIB1* transcript levels were compared with mock-treated plants at the same time point. Soybean *GmEF1β* was used as an internal control to normalize all data. Three biological replicates were averaged and statistically analysed using Student’s *t*-test (*P<0.05, **P<0.01). Bars indicate standard error of the mean. (D) *GmPIB1* mRNA levels in various soybean plant tissues. Leaves, roots, and stems were harvested from 14-day-old plants. The experiment was performed on three biological replicates, each with three technical replicates. Bars indicate standard error of the mean. (E) GUS histochemical staining analysis of *pGmPIB1:GUS*. *pGmPIB1:GUS* and *p35S:GUS* transgenic soybean hairy roots were produced by *A. tumefaciens*-mediated transformation and treated with 100 μM MeJA, 2 mM SA, or ET for 6 h. GUS histochemical staining results 3 h after treatment are shown compared with roots treated with water. Bars, 1 cm. (F) GUS activity analysis of *GmSPOD1* promoter expression. GUS activity was measured using a 4-methylumbelliferyl-D-glucuronide assay. The data represent the means ±SD of three independent experiments. (This figure is available in color at *JXB* online.)

To investigate the expression pattern of *GmPIB1* in response to phytohormone treatment, we performed qRT-PCR to examine *GmPIB1* transcript levels in ‘L77-1863’ soybean plants. *GmPIB1* expression was responsive to MeJA, ET, and SA treatment. *GmPIB1* mRNA levels rapidly increased under these treatments, reaching a maximum level at 6 h after treatment, followed by a rapid decline (Fig. 2A–C). In ‘L77-1863’ plants, *GmPIB1* was constitutively and highly expressed in stems, follow by roots and leaves (Fig. 2D). To elucidate the regulatory mechanism of *GmPIB1* under MeJA, ET, and SA treatment, we measured *GmPIB1* promoter activity in hairy roots at 6 h after treatment. GUS activity driven by the *GmPIB1* promoter (*pGmPIB1*) was weak under control (water) conditions, but it increased approximately 8- and 2.5-fold compared with the control under MeJA and SA treatment, respectively (Fig. 2E, F). These results suggest that GmPIB1 is primarily involved in the response to MeJA treatment.

### GmPIB1 is a transcriptional repressor that binds to the E-box sequence

**Fig. 3.**
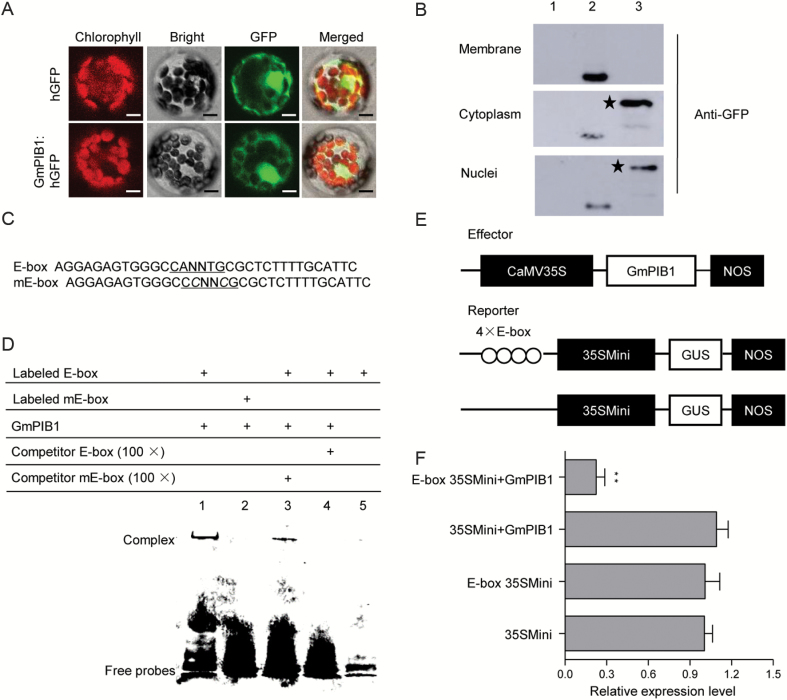
Sequence-specific binding activity of GmPIB1 to the E-box element. (A) Subcellular localization of GmPIB1–hGFP fusion protein. Subcellular localization was investigated in Arabidopsis protoplasts by confocal microscopy. The fluorescence from humanized GFP (hGFP) and the fusion protein GmPIB1–hGFP was observed under white light, UV light, and red light separately. Bars, 10 μm. (B) Immunoblot analysis detecting GmPIB1–hGFP fusion protein in the cytoplasm and nuclei. Line 1, Arabidopsis protoplasts (negative control); line 2, hGFP; line 3, GmPIB1–hGFP fusion protein. Anti-GFP was used to detect GmPIB1–GFP fusion protein in Arabidopsis cells. An asterisk denotes the specific band of the fusion protein GmPIB1–hGFP. (C) Nucleotide sequences of the E-box and mE-box probes. (D) EMSA showing sequence-specific binding of the recombinant GmPIB1 protein to the E-box. Lane 1, labeled E-box probe and GmPIB1 protein; lane 2, labeled mE-box probe and GmPIB1 protein; lane 3, titration using a cold mE-box sequence as a competitor; lane 4, titration using a cold E-box sequence as a competitor; lane 5, EMSA performed with only the free E-box probe. (E) Schematic diagram of the reporter and effector constructs. The reporter plasmids contained four repeats of the E-box sequence and 35Smini, and the effector plasmids encoded GmPIB1 under the control of the CaMV 35S promoter. (F) Relative GUS activity in transactivation assays. The effector and reporter plasmids were co-transfected into Arabidopsis protoplasts. The numbers show the fold increase in GUS activity compared with the vector E-box/35Smini promoter (E-box 35SMini) alone. The experiments were performed on three biological replicates and statistically analysed using Student’s *t*-test (**P<0.01). Bars indicate standard error of the mean. (This figure is available in color at JXB online.)

To investigate the subcellular localization of GmPIB1, we expressed a gene construct encoding GmPIB1–humanized GFP (hGFP) fusion protein under the control of the 35S promoter in Arabidopsis protoplasts. Confocal immunofluorescence and immunoblot analysis showed that hGFP alone was uniformly distributed throughout the cell, whereas transformed cells carrying GmPIB1–hGFP fusion protein localized to the cytoplasm and nuclei (Fig. 3A, B).

To express GmPIB1 in *Trans*etta (DE3) *E. coli* cells, we cloned the coding sequence of *GmPIB1* into pET-29b, an expression vector with a His-tag. Upon induction by IPTG, GmPIB1 was expressed as a major soluble protein product at 1, 2, and 4 h (Supplementary Fig. S3, lanes 2, 3, and 4). The molecular mass of the puriﬁed protein was approximately 21 kDa, as revealed by SDS-PAGE (Supplementary Fig. S3, lane 5), which is consistent with its calculated molecular mass (21.33 kDa). Immunoblotting of puriﬁed recombinant GmPIB1 protein conﬁrmed its speciﬁc immune reactivity to anti-His antibodies (Supplementary Fig. S3, lane 6).

To determine whether GmPIB1 binds to the *cis*-acting element of the E-box in its target promoters *in vitro*, we subjected purified His-tagged GmPIB1 to an EMSA with a digoxigenin-ddUTP-labeled double-stranded oligonucleotide E-box probe. The sequences of the E-box and mutated E-box (mE-box) are shown in Fig. 3C. When the E-box was used as a probe, GmPIB1 caused a mobility shift in labeled E-box probe (Fig. 3D, lane 1), which migrated more slowly than the free probe (Fig. 3D, lane 5). Furthermore, when mE-box was used in the assay, this mobility shift was not observed (Fig. 3D, lane 2). We conducted competition experiments to examine the specificity of the mobility shift. When the ratio of unlabeled-to-labeled E-box probe was 100:1, almost no labeled probe was bound (Fig. 3D, lane 4), and when 100-fold unlabeled mE-box probe was used as the competitor, no binding competition was observed (Fig. 3D, lane 3).

To investigate whether GmPIB1 is a transcriptional repressor, we performed a transactivation assay in Arabidopsis protoplasts using a reporter gene with four tandem copies of the E-box and effector plasmids with GmPIB1 (Fig. 3E). As shown in Fig. 3F, GmPIB1 appeared to repress reporter gene expression, since GUS expression was reduced to 71% of control levels in the presence of this protein. Overall, these results suggest that GmPIB1 is an E-box-specific DNA binding protein that acts as a transcriptional repressor in plant cells.

### GmPIB1 can form homodimers

**Fig. 4.**
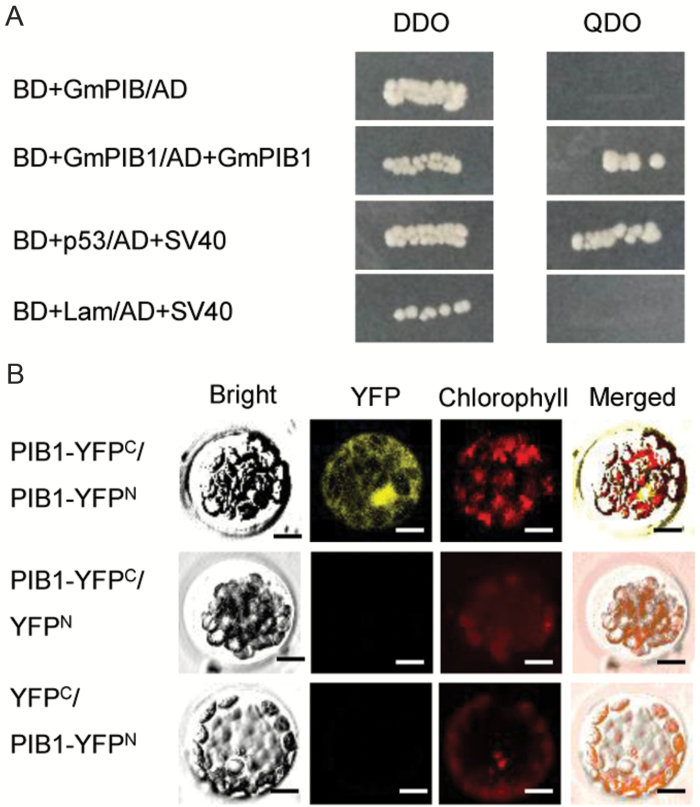
GmPIB1 forms a homodimer in yeast cells and *in planta*. (A) Yeast cells of strain Y_2_H harboring pGBKT7-GmPIB1 and pGADT7-GmPIB1 plasmid combinations were grown on either SD/−Trp/−Leu or SD/−Trp/−Leu/−His/−Ade medium. Yeast cells carrying the pGBKT7-53 and pGADT7-SV40 plasmids were used as the positive control; yeast cells harboring the pGBKT7-Lam and pGADT7-SV40 plasmids were used as the negative control. (B) BiFC analysis of the interaction of GmPIB1 with itself. GmPIB1–YFP^N^ and GmPIB1–YFP^C^ were co-transfected into Arabidopsis protoplasts. The bright-field, YFP fluorescence (yellow), chlorophyll autofluorescence (red), and combined images were visualized under a confocal microscope 16 h after transfection. Bars, 10 μm. (This figure is available in color at JXB online.)

The bHLH TFs form homodimers or heterodimers, which is a prerequisite for DNA binding, and each partner binds to half of the DNA recognition sequence (Ma *et al.*, 1994; Shimizu *et al.*, 1997; Feller *et al.*, 2011). To determine whether GmPIB1 forms homodimers in yeast cells, we fused full-length *GmPIB1* to the DNA-binding domain of GAL4 (BD) (Clontech, Palo Alto, CA, USA) and subjected it to a transcriptional activation activity by growing the yeast cells on SD/–Leu/–Trp (DDO) and SD/–Ade/–His/–Leu/–Trp (QDO) media. Together with the GAL4 activation domain (AD), yeast cells carrying full-length *GmPIB1* fused to the GAL4 DNA binding domain grew on DDO, but not on QDO medium (Fig. 4A). Further analysis suggested that in yeast cells carrying BD-GmPIB1 and AD-GmPIB1, the transcription of downstream reporter genes was activated, and the cells grew on QDO medium (Fig. 4A).

To further confirm the occurrence of these interactions *in planta*, we performed a bimolecular fluorescence complementation (BiFC) assay involving transient expression in Arabidopsis protoplasts. Co-expression of both N-terminal yellow fluorescent protein (YFP^N^)-tagged GmPIB1 and C-terminal YFP (YFP^C^)-tagged GmPIB1 resulted in significant fluorescence in the chloroplasts of Arabidopsis protoplasts (Fig. 4B). However, no fluorescence was detected in Arabidopsis protoplasts co-transformed with YFP^N^–GmPIB1 and YFP^C^ or YFP^C^–GmPIB1 and YFP^N^. These results suggest that GmPIB1 interacts with itself *in planta*.

### Expression of *GmPIB1* in soybean hairy root affects ROS levels

**Fig. 5.**
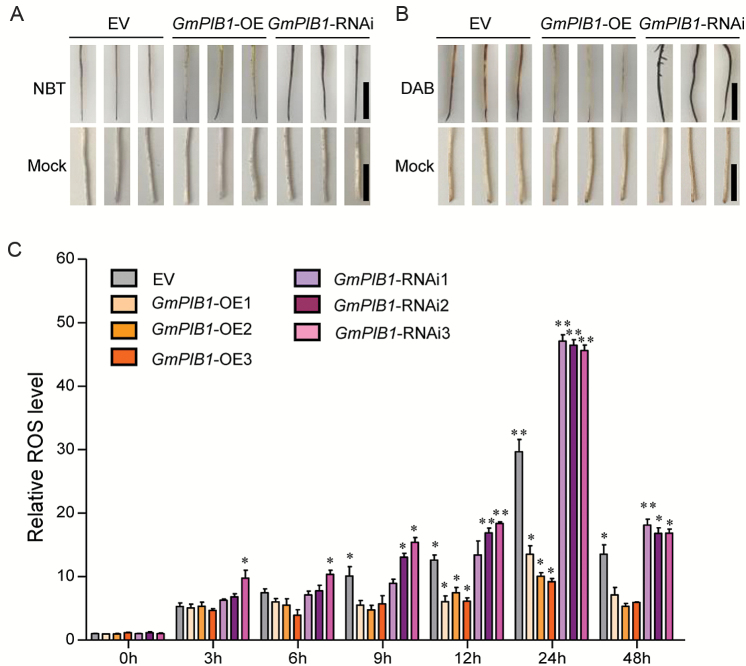
Analysis of ROS levels in *GmPIB1*-OE, *GmPIB1*-RNAi, and EV transgenic soybean hairy roots. (A) NBT staining of O_2_^−^ in 20-day-old EV, *GmPIB1*-OE, and *GmPIB1*-RNAi soybean hairy roots after *P. sojae* zoospore treatment for 48 h. Bars, 1 cm. (B) DAB staining of H_2_O_2_ in 20-day-old EV, *GmPIB1*-OE, and *GmPIB1*-RNAi soybean hairy roots under *P. sojae* zoospore treatment for 48 h. Bars, 1 cm. (C) Relative ROS levels in EV, *GmPIB1*-OE1, *GmPIB1*-OE2, *GmPIB1*-OE3, *GmPIB1*-RNAi1, *GmPIB1*-RNAi2, and *GmPIB1*-RNAi3 soybean hairy roots at 0, 3, 6, 12, 24, and 48 h after *P. sojae* infection. Relative ROS levels were measured, i.e. the ratio of total ROS levels in soybean hairy roots treated with *P. sojae* zoospores versus that in hairy roots treated with equal amounts of sterile water (mock) at the same time point. Three biological replicates, each with three technical replicates, were averaged and statistically analysed using Student’s *t*-test (*P<0.05, **P<0.01). Bars indicate standard error of the mean. (This figure is available in color at *JXB* online.)

ROS are key signaling molecules that are produced in response to biotic and abiotic stress and trigger a variety of plant defense responses (Hückelhoven and Kogel, 2003; Soosaar *et al.*, 2005; Takabatake *et al.*, 2007; Shetty *et al.*, 2008; Perez and Brown, 2014). H_2_O_2_ and superoxide (O_2_^−^) are the primary ROS components (Mittler *et al.*, 2004; Foyer and Shigeoka 2011). We therefore compared ROS production in EV, *GmPIB1*-OE, and *GmPIB1*-RNAi hairy roots after *P. sojae* zoospore inoculation by *in situ* NBT staining of superoxide anions and DAB staining of H_2_O_2_. Upon infection with *P. sojae* zoospores, we observed a dramatic increase in superoxide anion and H_2_O_2_ contents in EV hairy roots at 48 h after inoculation (Fig. 5A, B). Compared with EV hairy roots, lower levels of superoxide anion and H_2_O_2_ were detected in *GmPIB1*-OE roots, whereas higher levels were detected in *GmPIB1*-RNAi roots (Fig. 5A, B). We also measured relative ROS levels in EV, *GmPIB1-OE*, and *GmPIB1*-RNAi transgenic hairy roots at 0, 3, 6, 12, 24, and 48 h after incubation with *P. sojae*. The relative ROS levels gradually increased in EV, *GmPIB1*-OE, and *GmPIB1*-RNAi with increasing incubation time (Fig. 5C) and were signiﬁcantly lower in the *GmPIB1*-OE lines and significantly higher in the *GmPIB1*-RNAi lines compared with EV hairy roots at the same time point (Fig. 5C). These results suggest that overexpressing *GmPIB1* efficiently reduces ROS accumulation in soybean.

### GmPIB1 represses the expression of *GmSPOD1* in transgenic soybean hairy roots

**Fig. 6.**
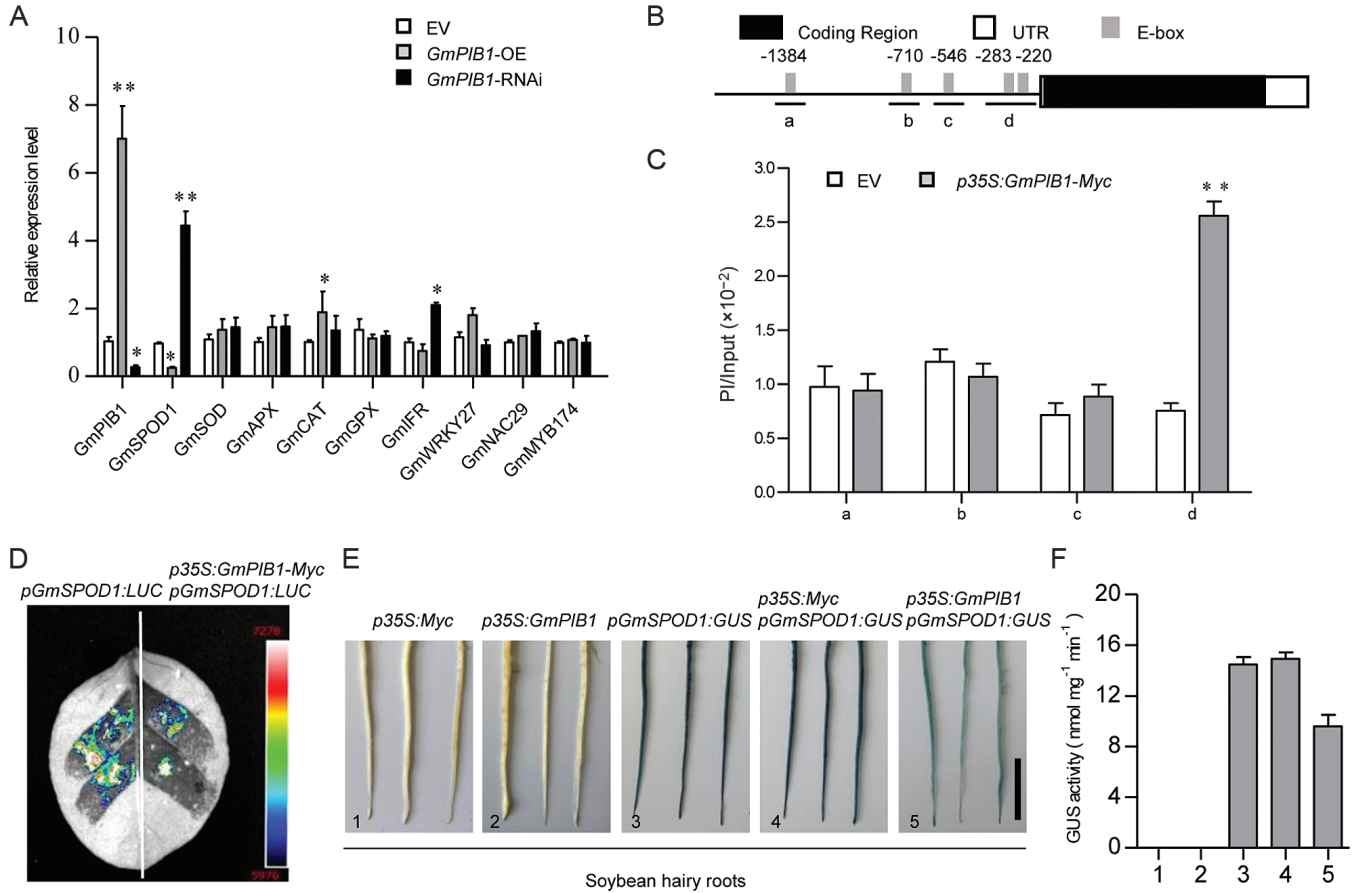
Analysis of ROS-induced gene expression in GmPIB1 transgenic and EV soybean hairy roots. (A) GmPIB1-modulated gene expression in *GmPIB1*-OE and *GmPIB1*-RNAi hairy roots compared with EV, as revealed by qRT-PCR. Soybean *GmEF1β* was used as an internal control to normalize all data. (B, C) ChIP analysis of GmPIB1 binding to the *GmSPOD1* promoter in *GmPIB1-Myc* transgenic soybean hairy roots and EV. Chromatin from *GmPIB1*-*Myc* transgenic and EV hairy roots was immunoprecipitated with anti-Myc antibody and treated without antibodies. The precipitated chromatin fragments were analysed by qPCR using four primer sets amplifying four regions upstream of *GmSPOD1* (*GmSPOD1a*, *GmSPOD1b*, *GmSPOD1c*, *GmSPOD1d*), as indicated. One-tenth of the input (without antibody precipitation) of chromatin was analysed and used as a control. Three biological replicates, each with three technical replicates, were averaged and statistically analysed using Student’s *t*-test (*P<0.05, **P<0.01). Bars indicate standard error of the mean. (D) GmPIB1 represses *GmSPOD1* promoter activity in *N. benthamiana* leaves. *Agrobacterium tumefaciens* GV3101 strains harboring *pGmSPOD1:LUC* and *p35S: GmPIB1* were transfected into *N. benthamiana* leaves. Luciferase imaging was performed 72 h after injection. (E) GmPIB1 represses *GmSPOD1* promoter activity in soybean hairy roots. *Agrobacterium rhizogenes* K599 strains harboring *p35S: GmPIB1*, and *pGmSPOD1:GUS* were transfected into soybean hairy roots. Line 1, *pGmSPOD1:GUS*; line 2, *p35:Myc*; line 3, *p35S: GmPIB1-Myc*; line 4, *p35:Myc* and *pGmSPOD1:GUS*; line 5, *p35S:GmPIB1-Myc* and *pGmSPOD1:GUS*. (F) GUS activity analysis of *GmSPOD1* promoter expression. GUS activity was measured using a 4-methylumbelliferyl-D-glucuronide assay. The *x*-axis numbers correspond to the numbers 1–5 in (E). The data represent the means ±SD of three independent experiments. (This figure is available in color at *JXB* online.)

To address how GmPIB1 affects ROS generation, we performed qRT-PCR in EV, *GmPIB1*-OE, and *GmPIB1*-RNAi hairy roots to measure the relative expression of genes that are known to take part in ROS production, such as the peroxidase gene *GmSPOD1* (NM_001252802); the ascorbate peroxidase gene *GmAPX* (L10292.1); the catalase gene *GmCAT* (AK286272.1); the superoxide dismutase gene *GmSOD* (XM_003526765.3); the glutathione peroxidase gene *GmGPX* (XM_006600055.2); the TF genes *GmNAC29* (XM_003556741), *GmWRKY27* (DQ322695), and *GmMYB174* (DQ822939); and the isoﬂavone reductase gene *GmIFR* (NM_001254100). *SPOD1* was signiﬁcantly down-regulated in *GmPIB1*-OE hairy roots but markedly up-regulated in the *GmPIB1*-RNAi lines compared with EV (Fig. 6A). *GmCAT* was up-regulated in *GmPIB1*-OE hairy roots, and *GmIFR* was up-regulated in *GmPIB1*-RNAi lines, compared with the EV control. However, the expression of the other genes was not affected in *GmPIB1*-OE or *GmPIB1*-RNAi hairy roots versus the control (Fig. 5A).

Using the PLACE program (Higo *et al.*, 1999), we detected five E-box *cis*-elements in the 1.761-kb region upstream of the *GmSPOD1* promoter (Fig. 6B). To further determine the binding capacity of GmPIB1 to the promoter of *GmSPOD1*, we performed a ChIP-qPCR assay to compare the relative enrichment of specific *GmSPOD1* sequences in *GmPIB1*-OE and EV hairy roots using anti-Myc antibodies. GmPIB1 protein was highly enriched in the *GmSPOD1* promoter *d* site in the *GmPIB1*-OE lines, whereas it was present at extremely low levels in the EV control (Fig. 6C).

To further examine the regulatory effect of GmPIB1 on the expression of its target gene, we performed transient expression assays using 1.761 kb of the *GmSPOD1* promoter fused to *GUS* or *LUC* as a reporter (*pGmSPOD1:GUS* or *pGmSPOD1:LUC*). The effector construct harbored *GmPIB1* expressed under the control of the 35S promoter (*p35S:GmPIB1-Myc*). We transformed the reporter construct (*pGmSPOD1:LUC*) and the effector construct (*p35S:GmPIB1-Myc*) into healthy *N. benthamiana* leaves, finding that GmPIB1 significantly repressed the expression of *GmSPOD1* (Fig. 6D). When we transformed the reporter construct (*pGmSPOD1:GUS*) and the effector construct (*p35S:GmPIB1-Myc*) into soybean hairy roots, we detected GUS activity driven by the *GmSPOD1* promoter (Fig. 6Ea, F), but not by *p35S:Myc* (Fig. 6Eb, F) or *p35S:GmPIB1-Myc* (Fig. 6Ec, F). GmPIB1 significantly repressed the expression of *GmSPOD1* (Fig. 6Ee, F), whereas there was no change in expression when *pGmSPOD1:GUS* and *p35S:Myc* were co-transformed into hairy roots (Fig. 6Ed, F). Taken together, these findings strongly support the idea that GmPIB1 directly inhibits the expression of the downstream *GmSPOD1* gene.

### GmSPOD1 also functions in responses to *P. sojae* infection

**Fig. 7.**
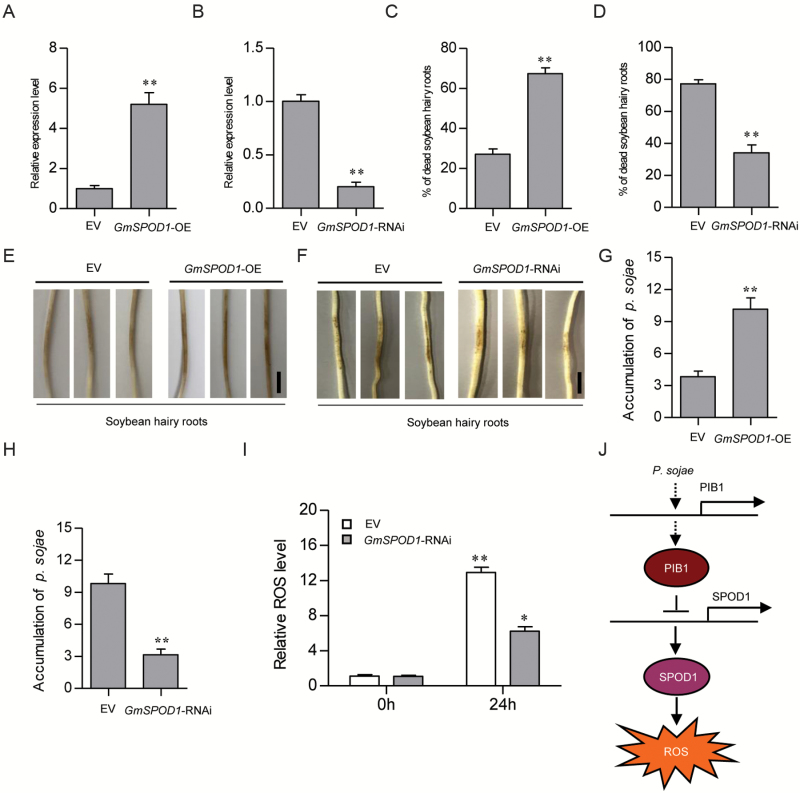
Knockdown of *GmSPOD1* increases resistance to *P. sojae*. (A, B) qRT-PCR analysis of *GmSPOD1* expression in EV, *GmSPOD1*-OE, and *GmSPOD1*-RNAi transgenic lines. (C, D) Percentage of dead hairy roots in EV, *GmSPOD1*-OE, and *GmSPOD1*-RNAi lines after *P. sojae* infection for 5 d. Each experiment contained at least 50 roots per line, and hairy roots were scored as dead when they were completely rotten. (E, F) Infection phenotypes of *GmSPOD1*-OE, *GmSPOD1*-RNAi, and EV soybean hairy roots after *P. sojae* inoculation for 2 d. (G, H) qRT-PCR analysis of relative *P. sojae* biomass based on the transcript level of *P. sojae TEF1*. (I) Relative ROS levels in EV versus *GmSPOD1*-RNAi lines at 0 and 24 h after *P. sojae* infection. Three biological replicates, each with three technical replicates, were averaged and statistically analysed using Student’s *t*-test (**P<0.01). Bars indicate standard error of the mean. (J) Model of the GmPIB1-mediated response to *P. sojae*. *GmPIB1* expression is induced by *P. sojae*. GmPIB1 inhibits *GmSPOD1* transcription by binding to the E-box element in its promoter. The suppression of *GmSPOD1* expression leads to decreased intracellular ROS levels. (This figure is available in color at *JXB* online.)

We then explored the possible role of GmSPOD1 in the response to *P. sojae* infection by analysing the phenotypes of EV, *GmSPOD1*-RNAi, and *GmSPOD1*-OE hairy roots after incubation with *P. sojae* zoospores. First, we tested *GmSPOD1*-OE and *GmSPOD1*-RNAi transgenic hairy roots using qRT-PCR (Fig. 7A, B). We then selected transgenic hairy roots and investigated their resistance to *P. sojae*. As shown in Fig. 7C, ~27% of inoculated EV hairy roots were completely dead and only ~67% of inoculated *GmSPOD1*-OE transgenic soybean hairy roots were completely dead at 5 d of incubation in resistant cultivar ‘L77-1863’. However, ~77% of inoculated EV hairy roots were completely dead and only ~35% of inoculated *GmSPOD1*-RNAi transgenic hairy roots were completely dead at 5 d of incubation in susceptible cultivar ‘Williams’ (Fig. 7D). After 2 d of incubation with *P. sojae* zoospores, all three *GmSPOD1*-OE transgenic hairy root lines exhibited enhanced wilting symptoms and chlorosis (Fig. 7E), whereas the *GmSPOD1*-RNAi lines displayed almost no visible lesions compared with the EV control (Fig. 7F).

We also analysed the relative biomass of *P. sojae* in infected hairy roots after 2 d of incubation with *P. sojae* zoospores. The biomass of *P. sojae* (based on *P. sojae TEF1* (GenBank accession no. EU079791) transcript levels) was significantly (*P*<0.01) higher in the roots of *GmSPOD1*-OE plants versus the EV control (Fig. 7G). The biomass of *P. sojae* was significantly (*P*<0.01) lower in the roots of *GmSPOD1*-RNAi plants compared with EV (Fig. 7H). Finally, we measured relative ROS levels in EV and *GmSPOD1*-RNAi transgenic hairy roots at 0 and 24 h after incubation with *P. sojae*. Relative ROS levels gradually increased with increasing inoculation time in both EV and *GmSPOD1*-RNAi plants (Fig. 7I). However, the relative ROS levels were signiﬁcantly lower in *GmSPOD1*-RNAi roots than in EV roots at the same time point (Fig. 7I). These results indicate that repressing *GmSPOD1* expression in soybean hairy roots improves resistance to *P. sojae*.

## Discussion

A bHLH TF gene was previously found to be up-regulated in all 10 *Rps* NILs examined under *P. sojae* treatment, as revealed by RNA-seq (Lin *et al.*, 2014). In this study, we determined that the bHLH TF designated GmPIB1 plays a crucial role in the response of soybean to *P. sojae* infection. Consistent with this finding, we found that *GmPIB1* transcript levels were much higher in the *P. sojae*-resistant soybean cultivar ‘L77-1863’ than in the susceptible cultivar ‘Williams’ (Fig. 1A, B). Under *P. sojae* treatment, *GmPIB1* was signiﬁcantly up-regulated in ‘L77-1863’ but not in ‘Williams’ (Fig. 1E). We also compared the gene and promoter sequences of *GmPIB1* between ‘Williams’ and ‘L77-1863’, finding no difference. Perhaps the difference in *GmPIB1* expression levels between the two cultivars is due to differences in *Rps*-mediated defense pathways. To date, a number of genes involved in *P. sojae* infection have been identified in soybean (Xu *et al.*, 2014; Cheng *et al.*, 2015; Dong *et al.*, 2015; Fan *et al.*, 2015, 2017; Jiang *et al.*, 2015; Yan *et al.*, 2016; Jing *et al.*, 2016; Zhao *et al.*, 2017). For example, in *GmERF5*-overexpressing soybean plants, *PR10*, *PR1-1*, and *PR10-1* are up-regulated and *P. sojae* resistance is significantly enhanced compared with wild type (Dong *et al.*, 2015). *GmIFR* encodes a NAD(P)H-dependent oxidoreductase and enhances resistance to *P. sojae* when overexpressed in soybean plants (Cheng *et al.*, 2015). Moreover, *GmBips*, which are targets of the *P. sojae* RxLR effector, negatively regulate plant defense responses against *P. sojae* infection (Jing *et al.*, 2016). Although some genes were shown to be involved in *P. sojae* responses, little is known about the biological functions of bHLH family members in soybean. To explore the molecular function of GmPIB1 in the response to *P. sojae*, we overexpressed *GmPIB1* in transgenic soybean hairy roots. These hairy roots exhibited significantly increased resistance to *P. sojae*, whereas resistance to *P. sojae* was compromised in *GmPIB1*-RNAi transgenic hairy roots compared with the control (Fig. 1H–M). These results indicate that GmPIB1 plays an important role in defense responses to *P. sojae* in soybean.

Plants encounter many environmental stresses in their natural environments and have evolved a wide range of mechanisms to cope with these stresses (Dixon and Paiva, 1995; Zhang *et al.*, 2008). When plants are overcome by certain pathogens, they recruit an inducible defense system to limit further pathogen ingression. The phytohormones SA, JA, and ET play central roles in biotic stress signaling following pathogen infection (Pieterse *et al.*, 2009; Robert-Seilaniantz *et al.*, 2011; Sugano *et al.*, 2013). The transcriptional cofactor NPR1 plays a key role in the SA-signaling pathway in several plant species (Vlot *et al.*, 2009). ERF1 plays a crucial role in ET-mediated disease resistance (Berrocal-Lobo *et al.*, 2002). ERF1 also regulates other hormone responses, particularly the JA-mediated defense response (Lorenzo *et al.*, 2003). ET and JA mediate defense responses against pathogen attack (partly) by inducing the expression of defense genes such as *PLANT DEFENSIN1.2* (*PDF1.2*). In the current study, we analysed the expression of *GmPIB1* following various hormone treatments (Fig. 2A–C) and determined that GmPIB1 might be primarily involved in responses to MeJA treatment.

The bHLH TFs play important roles in stress responses, which they mediate by binding to the E- and G-boxes present in the promoters of stress-related genes (Qian *et al.*, 2007; Liu *et al.*, 2014). AtbHLH122 specifically binds the E-box of the promoter regions of *CYP707A3* and represses its expression, thereby increasing ABA content to positively regulate drought, salt, and osmotic stress signaling in Arabidopsis (Liu *et al.*, 2014). PsGBF (a bHLH-type G-box binding factor) binds to the *PsCHS1* promoter and activates its expression to regulate the phenylpropanoid biosynthesis pathway in pea (Qian *et al.*, 2007). bHLH TFs also bind to the G- or E-box DNA motif to regulate plant development (Meng *et al.*, 2013; Liu *et al.*, 2013). For example, GmCIB1 (for cryptochrome-interacting bHLH1) interacts with the E-box-containing promoter sequence of *WRKY53b* to mediate light-induced regulation of leaf senescence in soybean (Meng *et al.*, 2013). In the current study, we demonstrated that GmPIB1 is localized to the nucleus and cytoplasm and specifically binds to the E-box *in vitro* (Fig. 3A–D). We also found that GmPIB1 suppressed the basal transcription levels of a reporter gene in Arabidopsis protoplasts (Fig. 3E, F). These findings suggest that GmPIB1 acts as an E-box-mediated transcriptional repressor.

ROS such as H_2_O_2_ and O_2_^−^ act as signaling molecules to regulate plant responses to biotic stress (Mittler *et al.*, 2004; Foyer and Shigeoka 2011; Shigeoka and Maruta 2014). Therefore, we measured ROS levels in *GmPIB1*-OE, *GmPIB1*-RNAi, and EV soybean transgenic hairy roots. ROS levels were reduced in *GmPIB1*-overexpressing transgenic soybean hairy roots (Fig. 5C), suggesting that GmPIB1 improves resistance to *P. sojae*, possibly by affecting ROS levels. ROS are produced not only as by-products of primary metabolism but also by plasma membrane- or apoplast-localized oxidases, peroxidases, and some TFs (Suzuki *et al.*, 2011; Cheng *et al.*, 2015; Wang *et al*., 2105*a*; Zhang *et al.*, 2015, 2016; Noshi *et al.*, 2016). We therefore performed qRT-PCR in EV, *GmPIB1*-OE, and *GmPIB1*-RNAi soybean hairy roots to measure the relative expression of genes known to be responsible for ROS production. Among *GmPIB1*-modulated genes, the expression of *GmSPOD1*, encoding a key enzyme for ROS production, was down-regulated in hairy roots overexpressing *GmPIB1* and up-regulated in *GmPIB1* RNA interference lines (Fig. 6A). Using ChIP-qPCR analysis, we also demonstrated that GmPIB1 directly binds to the E-box within the *d* site region of the *GmSPOD1* promoter (Fig. 6B, C). These results suggest that GmPIB1 directly represses *GmSPOD1* expression by binding to the E-box in its promoter.

Some enzymes involved in *P. sojae* infection have been identified in soybean (Subramanian *et al.*, 2005; Graham *et al.*, 2007; Cheng *et al.*, 2015). For example, silencing of either isoflavone synthase or chalcone reductase genes led to the breakdown of resistance to race 1 *P. sojae* in soybean (Subramanian *et al.*, 2005; Graham *et al.*, 2007). In the current study, *GmSPOD1*-OE hairy roots showed increased susceptibility to *P. sojae*, whereas *GmSPOD1*-RNAi hairy roots showed increased resistance to this pathogen (Fig. 7E, F). These results indicate that the inhibition of *GmSPOD1* expression by GmPIB1 enhances resistance to *P. sojae* in soybean.

Based on our data, we propose a model for the pathway regulating the defense response against *P. sojae* infection in soybean (Fig. 7J). According to this model, the bHLH TF GmPIB1 is a positive regulator of the response to *P. sojae* infection. During *P. sojae* infection, *GmPIB1* transcription is activated and this TF binds to the promoter of *GmSPOD1*, thereby directly inhibiting its expression. Subsequently, the reduced expression of *GmSPOD1* leads to decreased intracellular ROS levels and enhanced resistance to *P. sojae* in soybean plants. Our findings provide important insights into the mechanism underlying the response of soybean to *P. sojae* infection and offer a strategy for designing and breeding *P. sojae*-resistant soybean by genetically manipulating a bHLH gene.

## Supplementary data

Supplementary data are available at JXB online.

Fig. S1. Nucleotide and amino acid sequences of GmPIB1 cDNA.

Fig. S2. Resistance analysis of GmPIB1 transgenic soybean hairy roots.

Fig. S3. Expression and puriﬁcation of fusion protein.

Table S1. List of primers used in this study.

Supplementary Figures and Tables

## Author contributions

PX, SZ, QC, and LD designed the research. QC, LD, TG, TL, NL, and LW performed the research. XC and JW analysed the data. PX, SZ, QC, and LD wrote the article.
