# Supplementary material for: The bHLH transcription factor GmPIB1 facilitates resistance to Phytophthora sojae in Glycine max
Source: J Exp Bot. 2018 Mar 22;69(10):2527–41. doi: 10.1093/jxb/ery103 (PMC5920285; doi:10.1093/jxb/ery103)
Supplement: Supplementary Figures and Tables [file ery103_suppl_supplementary_figures_and_tables.pdf]

The bHLH Transcription Factor GmPIB1 Facilitates Resistance to *Phytophthora sojae* in *Glycine max*

Qun Cheng[1, 2†], Lidong Dong[1, 2†], Tianjiao Gao[1†], Tengfei Liu[1†], Ninghui Li[1, 3†], Le Wang[1†], Xin Chang[1], Junjiang Wu[4], Pengfei Xu[1*] and Shuzhen Zhang[1*]

[1]Soybean Research Institute/Key Laboratory of Soybean Biology of Chinese Education Ministry, Northeast Agricultural University, Harbin 150030, China

[2]School of Life Sciences, Guangzhou University, Guangzhou 510006, China

[3]Jiamusi Branch Academy of Heilongjiang Academy of Agricultural Sciences, Jiamusi 154000, China

[4]Soybean Research Institute of Heilongjiang Academy of Agricultural Sciences, Key Laboratory of Soybean Cultivation of Ministry of Agriculture P. R. China, Harbin 150086, China

ORCID IDs: 0000-0002-3311-0092 (S.Z.)

Qun Cheng, E-mail: chengqun0118@126.com

Lidong Dong, E-mail: donglidong1021@126.com

Tianjiao Gao, E-mail: 1027766012@qq.com

Tengfei Liu, E-mail: lafite15@outlook.com

Ninghui Li, E-mail: lnh000@163.com

Le Wang, E-mail: 770207082@qq.com

Xin Chang, E-mail: 18246197122@163.com

Junjiang Wu, E-mail: nkywujj@126.com

Pengfei Xu, E-mail: xupengfei@neau.edu.cn

Shuzhen Zhang, E-mail: zhangshuzhen@neau.edu.cn

[†]contributed equally to this research

*correspondence author

Pengfei Xu, Tel:+86 451 55191487 E-mail: xupengfei@neau.edu.cn

Shuzhen Zhang, Tel:+86 451 55191487 E-mail: zhangshuzhen@neau.edu.cn

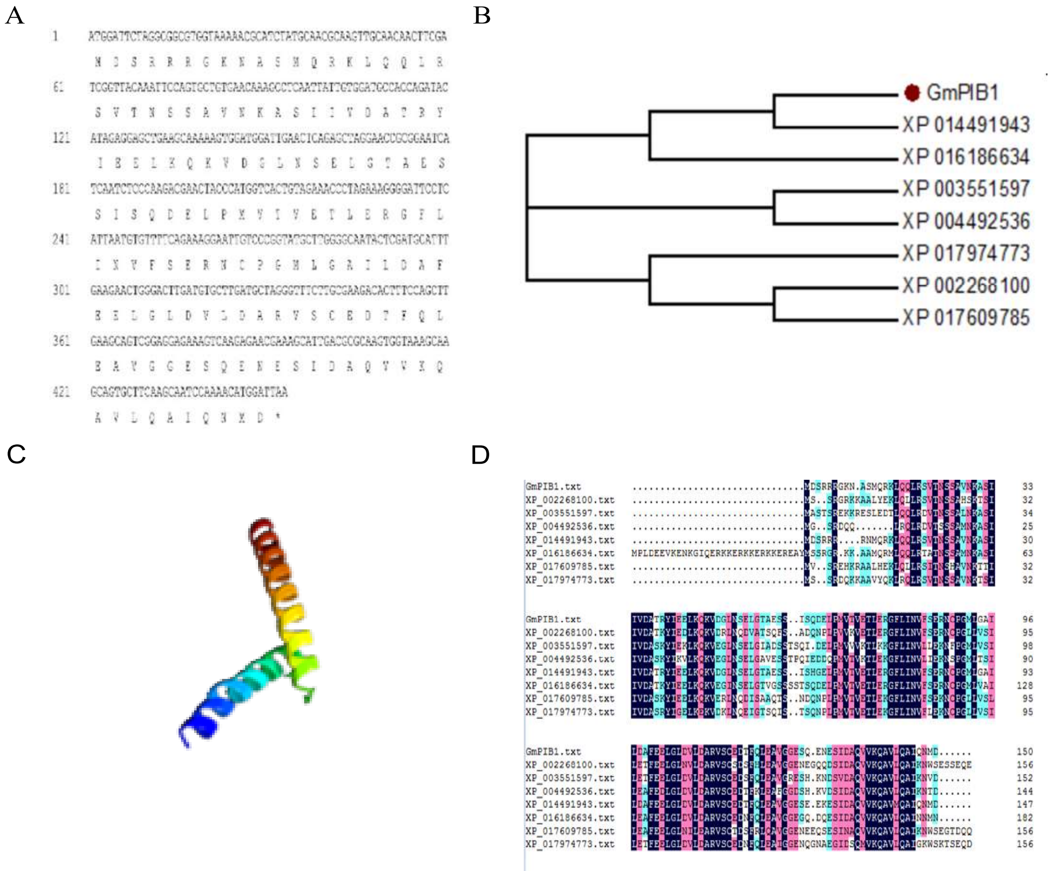

Figure S1. Nucleotide and amino acid sequences of GmPIB1 cDNA. A. Nucleotide and amino acid sequences of GmPIB1 cDNA. bHLH domain is underlined. B. Phylogenic analysis of *GmPIB1* compared with other bHLH transcription factors. The GenBank accession numbers are as follows: *Glycine max* bHLH transcription factor (XP_003551597), *Arachis ipaensis* bHLH transcription factor (XP_016186634), *Theobroma cacao* bHLH transcription factor (XP_017974773), *Vigna radiata* var. *radiata* bHLH transcription factor (XP_014491943), *Vitis vinifera* bHLH transcription factor (XP_002268100), *Gossypium arboretum* bHLH transcription factor (XP_017609785), and *Cicer arietinum* bHLH transcription factor (XP_004492536). C. The predicted three-dimensional structure of GmPIB1. D. Alignments of the amino acid sequence of GmPIB1 with other plant bHLH proteins.

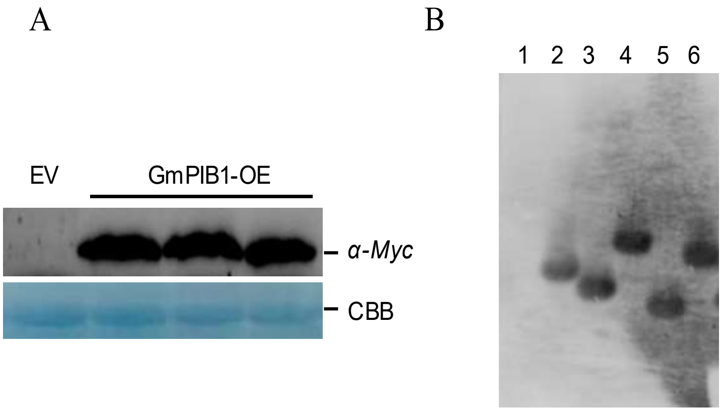

Figure S2. Resistance analysis of GmPIB1 transgenic soybean hairy roots. A. Immunoblots showing the expression of GmPIB1-Myc fusion protein in three independent *GmPIB1*-OE soybean hairy roots and the EV controls. The total protein extracts were analyzed in a 12% SDS-PAGE gel and probed with an anti-Myc antibody for the immunoblot. Coomassie Brilliant Blue (CBB) stain was used as a loading control. B. The *GmPIB1*-RNAi transgenic soybean hairy roots were tested using Southern blot. Lane 1, non-transformed soybean genomic DNA as a negative control; line 2, EV transformed soybean plants genomic DNA; lanes 3–6, *GmPIB1*-RNAi transformed soybean plants genomic DNA. Twenty micrograms of genomic DNA digested by the restriction enzyme Hind III was hybridized with the probe derived from the bar gene.

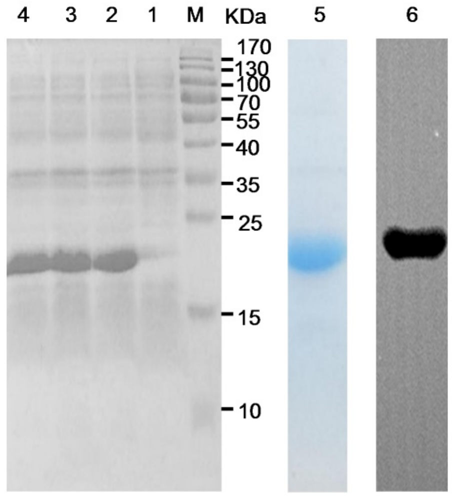

Figure S3. Expression and purification of fusion protein. After IPTG induction, *Trans*etta cells containing *pET29b-GmPIB1* were grown at 37 °C for 1, 2, or 4 h. Lane 1, protein of total cells without IPTG induction; lane 2, protein of total cells with IPTG induction for 1 h; lane 3, protein of total cells with IPTG induction for 2 h; lane 4, induction for 4 h; lane 5, recombinant GmPIB1 protein purified with Nickel-CL agarose affinity chromatography; M, molecular marker; Lane 6, western blotting of the purified recombinant GmPIB1 protein with an anti-His tag primary antibody probe.

Supplemental Table 1. List of Primers Used in This Study.

Primers for quantitative real-time PCR

**Primer name**	**Primers (5'-3')**
PIB1-qPCR-F	CATCAATCTCCCAAGACGAA
PIB1-qPCR-R	AGCACATCAAGTCCCAGTTC
IFR-qPCR-F	GAGGAGGATAGTGAGGGCAA
IFR-qPCR-R	CCTGATGTGAACACCAGAGATG
WRKY27-qPCR-F	AGGAACTGAAGCGGGTGA
WRKY27-qPCR-R	TTCGCAAAGTGTTGTAGTTCTC
SPOD-qPCR-F	AGACGGTGACGAGAAGCAAC
SPOD-qPCR-R	CGTGCTGCGATAACAAGGAT
SOD-qPCR-F	AACCACTGCCAACCAGGACC
SOD-qPCR-R	ATGCTCCCAAACATCAATACCAA
APX-qPCR-F	GCACTCTGCTGGAACCTTTG
APX-qPCR-R	TGTCAAGACCGTTGTTAGCG
CAT-qPCR-F	GATATTCTCTTATGCTGATTCACAA
CAT-qPCR-R	TTGACCTCCTCATCCCTGTG
NAC29-qPCR-F	TACCTTTGTAACCAAGCCACCTC
NAC29-qPCR-R	TGACACGGTTGCTCGGTTAG
GPX-qPCR-F	CACTCAAGCCAGAACAACCC
GPX-qPCR-R	GGTTTGTAGGGAAAGGGCAG
MYB174-qPCR-F	TTTTCGGAATGACTCTATTTGTAAC
MYB174-qPCR-R	ACGAATGTTTAGAGGTATCGGTTT
EF1 β-F	CCACTGCTGAAGAAGATGATGATG
EFI β-R	AAGGACAGAAGACTTGCCACTC

Primers for constructs in plant transformation

**Primer name**	**Primers (5'-3')**
GmPIB1-MycF	GAAGATCTATGGATTCTAGGCGGCGT
GmPIB1-*MycR*	GCACGTGATCCATGTTTTGGATTGCTTG
Gm*PIB1RNAi1-F*	CTCGAGGTCACTGTAGAAACCCTA
Gm*PIB1RNAi1-R*	GAGCTCACTTTCTCCTCCGACTGCT
Gm*PIB1RNAi2-F*	GGATCCGTCACTGTAGAAACCCTA
Gm*PIB1RNAi2-R*	TCTAGAACTTTCTCCTCCGACTGCT
GmSPOD1RNAi1-F	CTCGAGGAACCATCGAAACGGACCCA
GmSPOD1RNAi1-R	GAGCTCTCCTGGTCAGAAGTGAACACT
GmSPOD1RNAi2-F	GGATCCGAACCATCGAAACGGACCCA
GmSPOD1RNAi2-R	TCTAGATCCTGGTCAGAAGTGAACACT

Primers for GUS assays

**Primer name**	**Primers (5'-3')**
GmPIB1-PF	AAGCTTATTGGATTGGCAGTAGCGG
GmPIB1-PR	GGATCCTGTTTGTTTCCCTCACGACTAC

GmSPOD1PF	CAAGCTTAAGTTCTTTCTGGGGCTGGAG
GmSPOD1PR	TCTAGATTGAGAAGCCAATACAAGGGAA

Oligos for EMSA probes

**Primer name**	**Primers (5’-3’)**
E-box-F	AGGAGAGTGGGC*CANNTG*CGCTCTTTTGCATTC
E-box-R	GAATGCAAAAGAGCG*CANNTG*GCCCACTCTCCT
mE-box-F	AGGAGAGTGGGC*CC*NN*CG*CGCTCTTTTGCATTC
mE-box-R	GAATGCAAAAGAGCG*CGNNGG*GCCCACTCTCCT
GmPIB1-HISF	GGAATTCCATGGATTCTAGGCGGCGT
GmPIB1-HISR	GCTCGAGATCCATGTTTTGGATTGCTTG

Primers for ChIP assays

**Primer name**	**Primers (5’-3’)**
GmSPOD1-aF	F: TATTGATTTGTGAATGAGGTGAGA
GmSPOD1-aR	R: TAACCTCTTTCCCTACACCTAATC
GmSPOD1-bF	F: AACAATATTGGTGTAAATTATTGG
GmSPOD1-bR	R: TTTATGTGTAAGTTTCAACATTCC
GmSPOD1-cF	F: ATCGCTATAATCATACATGAAAAA
GmSPOD1-cR	R: ATTATTTTGTTTTTGACATGTGAA
GmSPOD1-dF	F: CGAAGACGCGTTGAAGTGT
GmSPOD1-dR	R: GATGGCTGCTATTGTTTATGG
GmSPOD1-eF	F: ACCAAACTGTCTCAAAGAACCA
GmSPOD1-eR	R: TAGCAATGACCGAGTCTAAAAC

Primers for constructs in Subcellular Lcalization assays

**Primer name**	**Primers (5’-3’)**
GmPIB1-GF	GAAGATCTCATGGATTCTAGGCGGCGT
GmPIB1-GR	GACTAGTATCCATGTTTTGGATTGCTTG

Primers for constructs in BiFC assays

**Primer name**	**Primers (5’-3’)**
GmPIB1-BF	GAATTCATGGATTCTAGGCGGCGTGGT
GmPIB1-BR	GGATCCAATCCATGTTTTGGATTGCT
